# Late-onset inferior alveolar nerve paresthesia two years after coronectomy associated with progressive root migration: a case report

**DOI:** 10.3389/froh.2026.1865940

**Published:** 2026-07-21

**Authors:** Berker Doganer, Sabir Majidov, Alp Saruhanoglu, Firat Selvi

**Affiliations:** 1 Department of Oral and Maxillofacial Surgery, Faculty of Dentistry, Istanbul University, Istanbul, Türkiye; 2 Institute of Graduate Studies in Health Sciences, Oral and Maxillofacial Diseases and Surgery PhD Program, Istanbul University, Istanbul, Türkiye

**Keywords:** case report, coronectomy, entrapment, inferior alveolar nerve, late-onset complication, mandibular third molar, paresthesia, root migration

## Abstract

Coronectomy is a nerve-sparing technique used to reduce the risk of inferior alveolar nerve (IAN) injury during removal of mandibular third molar (M3) in close proximity to the mandibular canal. This case describes a 45-year-old female with a history of an uneventful coronectomy of the right M3 performed 2 years earlier, currently presenting with a late-onset inferior alveolar nerve (IAN) paresthesia and discomfort at the same site. Intraoral examination showed partial exposure of the retained roots to the oral cavity and the clinical neurosensory examination confirmed Level A paresthesia of the right lower lip and chin, characterised by loss of light touch and directional discrimination. Follow-up imaging revealed approximately 2.4 mm of migration of the retained roots towards the oral cavity. In the absence of clinical or radiographic signs of infection, the radiographic findings strongly supported a mechanical etiology. A secondary surgical procedure was performed to section and remove the roots individually, preserving the nerve, resulting in complete sensory recovery within one month. This case highlights that late-onset sensory disturbance may still occur after an initially uneventful coronectomy, in cases having intimate root-canal relationships. Timely surgical management, when indicated, may result in complete neurological recovery, as observed in this patient.

## 1 Introduction

The surgical extraction of the mandibular third molars (M3) is a frequently performed procedure in the oral surgery practice, yet it carries complication risks such as the inferior alveolar nerve (IAN) injury (1–3). Such injuries may cause temporary or permanent hypoesthesia, paresthesia, or dysesthesia affecting the lower lip, chin, or gingivae. Although permanent sensory alterations are rare, they can significantly impact a patient's quality of life (4, 5).

Coronectomy, or intentional partial odontectomy, was introduced as an alternative method to complete extraction, in high-risk cases, to minimize IAN damage (1–3). The technique involves removing the crown while preserving vital roots *in situ*, thereby avoiding direct surgical trauma to the nerve. Indications for coronectomy include vital M3s without infection, where radiographic findings suggest close proximity or potential encroachment of the canal (4, 6).

Clinical outcomes have supported coronectomy as a reliable nerve-sparing procedure. Most reported complications occur in the early post-operative period and may include localized infection, dry socket, or transient sensory changes. Late complications are uncommon and typically related to root migration or rare instances of root exposure intraorally. Consequently, root exposure may lead to periodontal disease or pulpitis of the residual root resulting in pain, and necessitating a second surgical intervention (3, 7).

This report presents a case of a late-onset IAN paresthesia, occurring two years after an uneventful coronectomy procedure, plausibly associated with progressive root migration and evolving root-canal spatial relationships over time. While root migration and its radiographic sequelae have been described extensively, late-onset neurosensory disturbance in the absence of infection or intraoral root exposure represents an underappreciated presentation within the spectrum of coronectomy sequelae. To the authors’ knowledge, only a small number of case reports describe late-onset IAN paresthesia following coronectomy in association with progressive root migration (8, 9); the present case is distinguished by the combination of an uneventful initial procedure, a two-year asymptomatic interval, in the absence of active infection, and complete sensory recovery following secondary root retrieval. The objective is to describe the clinical and radiographic findings, surgical management, and outcome, and highlight the importance of reassessment of the evolving root-canal spatial relationships over time when late-onset symptoms develop after coronectomy.

## 2 Case report

### 2.1 Patient information and initial presentation

**Figure 1:**
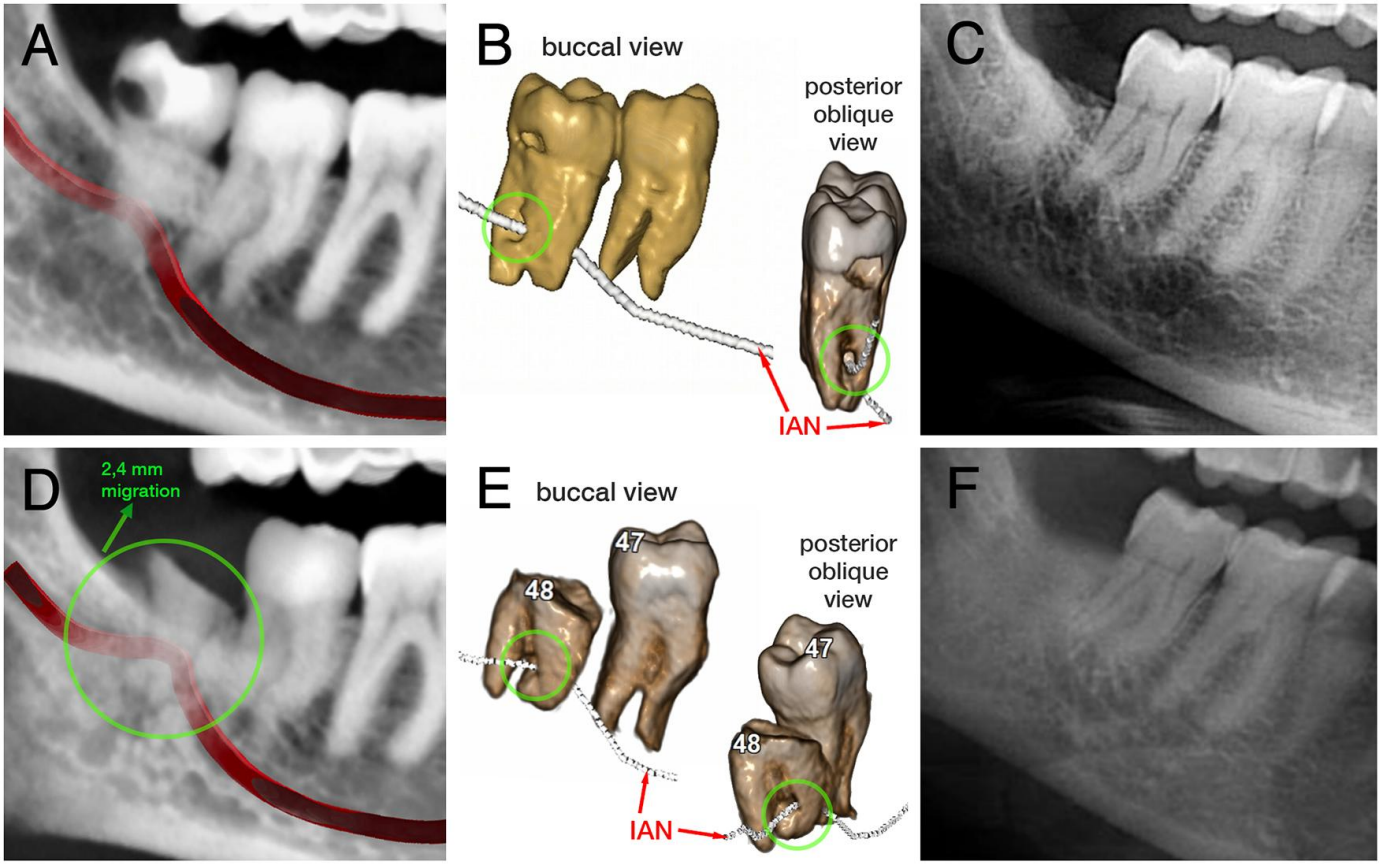
**(A)** Pre-operative reformatted panoramic image derived from CBCT data of right mandibular region, showing the mandibular canal trajectory (red). **(B)** CBCT-based segmentation demonstrating the relationship between the right M3, second molar and the inferior alveolar nerve (IAN); red arrow indicates the IAN course and green circles indicate the intimate root-IAN relationships; buccal view on the left and posterior oblique view on the right. **(C)** Immediate post-operative orthopantomography (OPG) after coronectomy of the right M3. **(D)** Two-year post-operative reformatted panoramic image reconstructed from CBCT data, the green circle and arrow demonstrate an estimated 2.4 mm of coronal migration of the retained roots and coronal displacement of the mandibular canal trajectory, consistent with presumed mechanical stretching of the IAN by the migrating root apices. **(E)** Two-year post-operative CBCT-based segmentation demonstrating the relationship between the right M3, second molar, and the inferior alveolar nerve (IAN); red arrow indicates IAN course and green circles indicate the intimate root-IAN relationship; buccal view on the left and posterior oblique view on the right. **(F)** One-year post-operative OPG, after secondary surgical removal of the retained roots, showing complete removal of the retained roots and absence of residual pathology.

A 45-year-old female patient initially presented to an outside facility with pain associated with a partially erupted right mandibular third molar (FDI 48). As per patient history, the tooth was decayed and symptomatic, with intermittent mild to moderate pain provoked by cold stimuli, but no spontaneous or nocturnal pain, consistent with a vital pulp. There were no signs of infection or swelling. The patient was otherwise healthy and reported no history of previous neurosensory disturbances. Initial panoramic radiography suggested close proximity in between the right M3 and the mandibular canal, prompting further three-dimensional evaluation with cone-beam computed tomography (CBCT). The radiographic progression from pre-operative assessment through root migration and post-operative recovery is documented in Figure 1 (Figures 1A–F). Further evaluation with CBCT demonstrated an unusual root morphology, with the apical portion of the fused roots encasing the IAN (Figures 1A,B). Due to the high risk of nerve injury, coronectomy was performed in that facility instead of extraction for this tooth (Figure 1C). Per patient's report, post-operative period was uneventful after the coronectomy with no neurosensory deficits.

### 2.2 Two-year follow-up and onset of new symptoms

Two years after the coronectomy, the patient returned with a new onset of increasing numbness and altered sensation in the right lower lip and chin region that had begun over the past few weeks. Thus, she was referred to our clinic, where further evaluation and management were performed.

**Figure 2:**
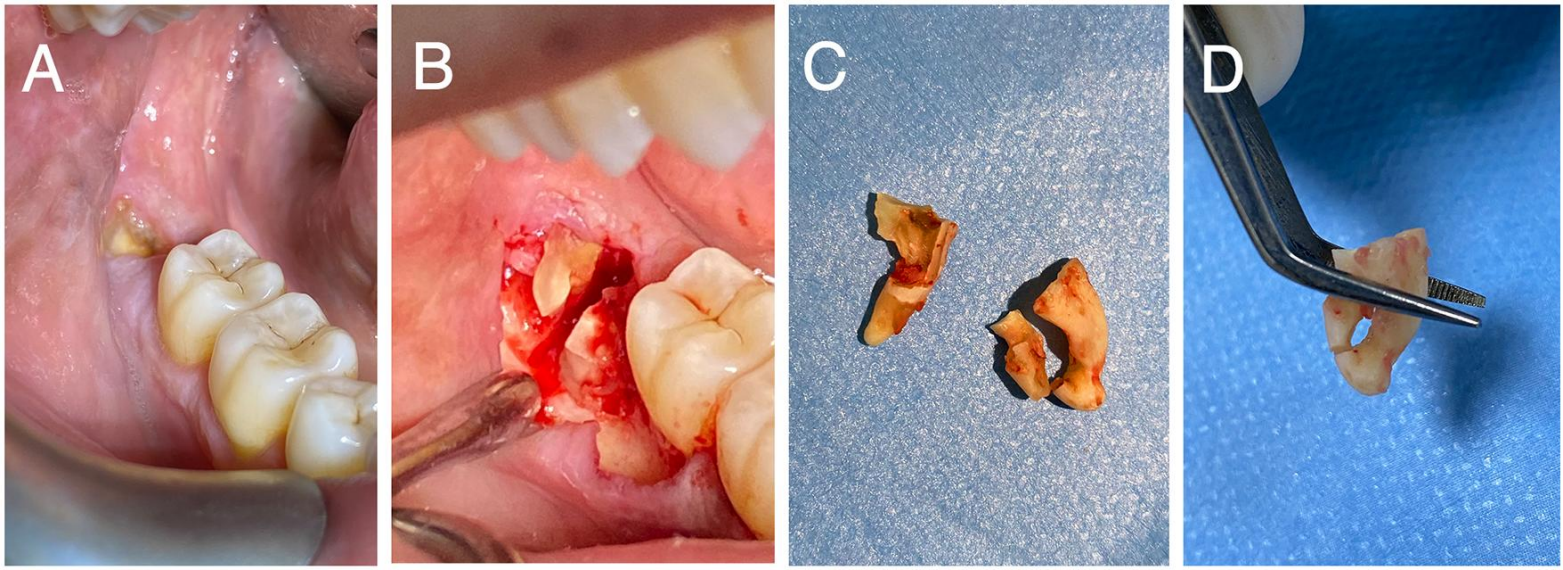
**(A)** Intraorally exposed root observed two years after the initial coronectomy. **(B)** Segmentation of the remaining M3 into mesial and distal root segments. **(C)** All extracted root fragments. **(D)** Close-up view of the retrieved mesial root fragment after sectioning, demonstrating the apical channel-like space between the fused mesial root components corresponding to the pathway of IAN observed in CBCT.

Intraoral examination showed the roots of the mandibular right third molar to be visible intraorally (Figure 2A). Clinical neurosensory assessment was performed using the mechanoreceptive and nociceptive tests described by Zuniga and Essick (10), and thus, paresthesia of the right lower lip and chin were confirmed. Among Level A tests, static light touch detection and directional brush discrimination impaired, while two point discrimination was partially preserved. Level B (contact detection) and Level C (pin pressure nociception and thermal discrimination) responses were intact, consistent with a Sunderland Grade I injury of the right inferior alveolar nerve (10, 11). A new CBCT scan demonstrated approximately 2.4 mm coronal migration of the retained roots, together with superior displacement of the mandibular canal trajectory in the direction of root eruption (Figures 1D,E). In the absence of clinical or radiographic signs of infection, the radiographic findings were considered consistent with a mechanical etiology for the late-onset paresthesia.

### 2.3 Second surgical intervention

A secondary operation was planned to relieve the presumed mechanical tension on the IAN by removing the migrated retained roots. Under local anesthesia [articaine hydrochloride 4% with 1:100,000 epinephrine (Maxicaine Fort; Vem, Turkey)], a standard envelope incision for M3 removal with anterior and posterior extensions was made to ease primary closure of the wound. A full thickness mucoperiosteal flap was then elevated to fully expose the retained root complex. Following minimal buccal trough preparation for access, root complex was sectioned gently using a H31L surgical fissure bur with a contraangle handpiece. The mesial and distal roots were initially sectioned in a buccal-lingual direction. Subsequently, the mesial root was initially further subdivided in a mesial-distal direction halfway using the bur and then the vertical division of the mesial root was completed by a gentle luxation using a periotome; this maneuver allowed atraumatic disengagement of the two mesial root segments from the IAN. Gentle retrieval of these mesial root segments were done using a small rongeur. During the removal of root segments, the inferior alveolar canal became exposed within the surgical field; the nerve was identified to be intact and it was carefully preserved. The surgical site was primarily closed with 4-0 resorbable sutures (Vicryl) (Figures 2A–D). The post-operative period was uneventful and within one month, the patient reported complete resolution of the neurosensory symptoms. At the one-year follow-up after secondary surgical intervention, she remained entirely asymptomatic, with normal sensation across all IAN distribution tests and no radiographic signs of residual pathology (Figure 1F). Table 1 summarizes the clinical chronological sequence of events from initial presentation to the final follow-up.

**Table 1:** Timeline of clinical events.

Timepoint	Event	Finding/action	Outcome
Year 0 (Day 0)	Initial presentation (outside facility)	Carious symptomatic partially erupted right M3; panoramic radiography -> intimate root-canal relationship; CBCT -> fused root apices encasing IAN	Coronectomy performed; root complex retained *in situ*
Year 0 (1–4 weeks)	Early post-operative period	Patient-reported uneventful healing	No neurosensory deficits
Year 2 (onset)	New symptom onset	Progressive numbness and altered sensation, right lower lip and chin; onset over preceding weeks	Patient referred to our department
Year 2 (assessment)	Clinical and radiographic evaluation	Partial intraoral root exposure; paresthesia of right lower lip and chin (loss of light touch and directional discrimination); CBCT - significant coronal root migration (2.4 mm), displacement of mandibular canal trajectory; no infection	Mechanical etiology strongly supported; secondary surgery planned
Year 2 (intervention)	Secondary surgical intervention	Staged root sectioning and retrieval under local anaesthesia (articaine HCl 4% with 1:100,000 epinephrine); IAN identified and preserved; 4-0 Vicryl primary closure	No intra-operative complications
Year 2 (+1 month)	Early post-operative follow-up	Neurosensory reassessment	Complete resolution of paresthesia reported
Year 3 (+1 year)	One-year follow-up	Normal neurosensory responses; OPG - no residual pathology	Fully asymptomatic; no further intervention

## 3 Discussion

Coronectomy has been widely accepted as a viable alternative to conventional extraction for mandibular M3s closely associated with the mandibular canal, with the primary objective of minimizing direct nerve injury. Numerous clinical studies and systematic reviews have demonstrated that coronectomy significantly reduces the incidence of immediate post-operative neurosensory disturbances when compared to total odontectomy (2, 3). The rationale behind this technique is based on preserving the apical root complex and avoiding manipulation of the neural sheath.

Root migration is a well-documented radiographic phenomenon after coronectomy and typically occurs within the first post-operative year, with most studies reporting average displacement between 2 and 4 mm (1, 7, 12–14). Kohara et al. (6) confirmed continued but minimal displacement beyond the first year, stabilizing around 3.4–3.5 mm by the second or third year. According to a recent systematic review, 46% of the coronectomy cases resulted in some amount of root migration (14). Similarly, Leung et al. (15) reported root movement even in cases with guided bone regeneration was performed after coronectomy. These findings indicate that migration is a physiological sequel rather than a complication and may contribute to a safer secondary operation for the roots’ removal, if ever required. Even in long-term follow-up studies extending up to ten years, most patients remain asymptomatic, and secondary operation is seldom required unless intraoral root exposure occurs (3). In our case, retrospective CBCT-based estimation of root migration was performed on multiplanar reformatted images obtained from baseline and two-year follow-up CBCT scans. After reorientation of both scans using the long axis of tooth 47 and the mandibular occlusal plane, vertical measurements were obtained on matched coronal sections from the mesial root apex, the distal root apex, and the most apical canal-related point within the mesial root to the corresponding lingual cortical reference point on the same section. Overall, an estimated 2.4 mm coronal root migration was identified.

Bone regeneration over retained roots is well documented, with high rates of coronal bone coverage reported across multiple long-term series, providing a barrier and potentially reducing further migration (3, 6). In general, technical principles of coronectomy include leaving the retained roots at least 3 mm below the crestal bone (1), and removing the residual enamel, as retained enamel remnants may impair ossification and soft tissue reattachment to enamel is not expected (4, 13). Reoperation after coronectomy is uncommon and typically related to root migration and exposure to the oral cavity rather than infection or nerve injury. Reported rates vary from 0.6% to 9%, depending on follow-up duration and inclusion criteria (2, 3, 6, 7, 13, 16). Leung and Cheung (16) documented the lowest incidence (0.6%), with a single case of persistent root exposure. Higher rates, such as 9.0% in the cohort reported by Kohara et al. (6), were associated with wound complications and root exposures. Notably, most secondary interventions occur after roots have migrated away from the mandibular canal, making delayed removal surgically safer than primary extraction.

A focused PubMed search using combinations of the terms “coronectomy” “root migration” “paresthesia”, “secondary surgery” and “inferior alveolar nerve entrapment” identified only a limited number of directly relevant case-based reports, supporting that delayed neurosensory complications related to post-coronectomy root migration remain sparsely documented. Steinberg and Nick (9) reported a closely related case in which post-coronectomy root migration displaced the IAN bundle and produced hypoesthesia; however, that case was associated with preceding pericoronitis, post-operative alveolar osteitis, and hook-shaped bifid root morphology, with symptom onset in the ninth post-operative month. Drage and Renton (8) also described an unusual retained-root case in which a perforated mandibular third molar root erupted together with the canal and was associated with recurrent paresthesia and infection years later. In contrast, the present case followed an initially uneventful coronectomy, showed no clear signs of active infection at the time of symptoms’ onset, and presented with new-onset paresthesia two years later, with complete sensory recovery after secondary root retrieval. Additional related but distinct reports include cases of mandibular third molars with unusual root morphology and pre-existing inferior alveolar nerve entrapment requiring staged surgical management, as well as intermittent IAN paresthesia associated with partially erupted M3, managed by coronectomy (17–19). These reports support the concept that intimate root-canal anatomy may become clinically significant in different ways and that altered root-canal relationships may produce neurosensory symptoms through stretching or compression of the IAN, supporting a mechanical traction-based explanation in the present case (9, 17, 18). However, they differ from the present case in that our patient remained asymptomatic for an extended post-operative period before developing late-onset symptoms in association with retained root migration.

This case underscores an important clinical consideration: although coronectomy is designed to prevent direct trauma to the nerve, it does not eliminate the potential for late-onset neural disturbance. Post-operative imaging obtained from the patient suggested that the retained roots were positioned relatively close to the crestal bone, below the recommended minimum of 3 mm to facilitate adequate ossification (1, 13). Nevertheless, the patient remained entirely asymptomatic for approximately 2 years post-operatively, suggesting that the paresthesia was related to the evolving root-canal spatial relationship over time rather than to the initial surgical outcome alone. The absence of symptoms during the initial post-operative period should not preclude long-term surveillance, particularly in cases with unusual root morphology or radiographic proximity to the canal. The prompt resolution of neurosensory symptoms after secondary root retrieval in this case further highlights the effectiveness of timely intervention when late complications arise.

Current evidence supports an immediate post-operative image and a routine panoramic review at approximately one year after coronectomy, with further imaging generally reserved for symptomatic presentations (1, 20). However, the present case, together with related reports of delayed nerve-related complications (9), suggests that patients with unusual root-canal anatomical relationships may benefit from at least annual clinical follow-up beyond the first post-operative year, even in the absence of routine additional imaging. Such visits allow directed questioning for subtle or intermittent sensory changes that patients may otherwise attribute to transient causes and not spontaneously report, as well as intraoral inspection of the coronectomy site. In such cases, pre-operative counselling should include the possibility of late-onset neurosensory symptoms, and any new sensory complaint or root exposure identified at review should prompt timely radiographic reassessment. While the overall safety profile of coronectomy remains favorable, clinicians should remain vigilant for delayed complications, even after initially successful outcomes.

Several limitations of this report should be acknowledged. As a single retrospective case, generalizability is inherently limited. The initial coronectomy was performed at an outside institution; the early post-operative course was based solely on patient report. The CBCT-based root migration measurement should be interpreted with caution, as precise standardization is difficult and even minimal landmark deviation and slight CBCT related distortion distortion may affect the quantitative migration assessment.

## 4 Conclusion

Root migration after coronectomy is a common phenomenon; however, neurosensory disturbances related to root migration appear to be rarely reported in the literature. This case demonstrates that late-onset IAN paresthesia may develop following coronectomy even after an initially asymptomatic post-operative course, particularly in patients with unusual anatomical root-canal relationships such as root encasement of the nerve. In such cases, patients should be informed pre-operatively about the possibility of late-onset neurosensory complications. When symptoms arise, prompt clinical and radiographic assessment, followed by timely surgical management when indicated, may result in complete neurological recovery, as observed in this patient.

## 5 Patient perspective

The patient reported that while the numbness in her lower lip and chin caused some disruption to her daily comfort—particularly during eating and speaking—it did not prevent her from carrying out daily activities. She described a gradual but steady improvement in sensation following the secondary procedure, with complete resolution of symptoms within one month. At her one-year follow-up, she remained entirely asymptomatic and expressed satisfaction with the outcome of the treatment.

## Data Availability

The original contributions presented in the study are included in the article, further inquiries can be directed to the corresponding author.
